# Bumblebee Pupae Contain High Levels of Aluminium

**DOI:** 10.1371/journal.pone.0127665

**Published:** 2015-06-04

**Authors:** Christopher Exley, Ellen Rotheray, David Goulson

**Affiliations:** 1 The Birchall Centre, Lennard-Jones Laboratories, Keele University, Stoke-on-Trent, Staffordshire, ST5 5BG, United Kingdom; 2 Evolution, Behaviour & Ecology, School of Life Sciences, University of Sussex, Brighton, BN1 9QG, United Kingdom; University of Guelph, CANADA

## Abstract

The causes of declines in bees and other pollinators remains an on-going debate. While recent attention has focussed upon pesticides, other environmental pollutants have largely been ignored. Aluminium is the most significant environmental contaminant of recent times and we speculated that it could be a factor in pollinator decline. Herein we have measured the content of aluminium in bumblebee pupae taken from naturally foraging colonies in the UK. Individual pupae were acid-digested in a microwave oven and their aluminium content determined using transversely heated graphite furnace atomic absorption spectrometry. Pupae were heavily contaminated with aluminium giving values between 13.4 and 193.4 μg/g dry wt. and a mean (SD) value of 51.0 (33.0) μg/g dry wt. for the 72 pupae tested. Mean aluminium content was shown to be a significant negative predictor of average pupal weight in colonies. While no other statistically significant relationships were found relating aluminium to bee or colony health, the actual content of aluminium in pupae are extremely high and demonstrate significant exposure to aluminium. Bees rely heavily on cognitive function and aluminium is a known neurotoxin with links, for example, to Alzheimer’s disease in humans. The significant contamination of bumblebee pupae by aluminium raises the intriguing spectre of cognitive dysfunction playing a role in their population decline.

## Introduction

There is on-going debate as to the causes and extent of declines of bees and other pollinators, with a growing consensus that pollinators are subject to a number of interacting stressors, including exposure to pesticides, infection with native and emerging pathogens, and declining abundance and diversity of floral resources [1–3]. Aside from pesticides, little attention has been paid to quantifying exposure to or impacts of other pollutants [4].

The most significant environmental contaminant of recent times is the metal aluminium [5]. Human activities such as the burning of fossil fuels resulting in ‘acid rain’, intensive agriculture producing acid sulphate soils and the mining of aluminium ores to make aluminium metal and salts have all contributed to the burgeoning biological availability of this non-essential metal [6]. Fish, trees, arable crops and humans are all impacted by aluminium and recent evidence suggests, at least, that bees are not immune to its increasing prevalence in the biotic cycle. For example, while there are very few data it has been shown that pollen is heavily contaminated with aluminium with analyses from Brazil indicating a mean content of 96μg/g [7]. Recent research has suggested that nectar may also be contaminated with aluminium and in experiments where nectar was replaced with a sugar solution spiked with aluminium bumblebees continued to forage and ingest this potentially toxic resource [8].

However, we do not know how commonly bees are exposed to aluminium, and no studies have investigated whether such exposure may contribute to bee health problems. Here, we quantify the concentration of aluminium in bumblebee pupae taken from colonies that had been foraging naturally in the UK landscape. We also examine whether aluminium concentration correlates with measures of colony fitness.

## Materials and Methods

### Sample collection

In May 2013, 20 commercially produced *Bombus terrestris audax* colonies were acquired from Biobest NV. Belgium. Each colony was numbered randomly upon arrival, the biogluc (glucose solution) bottles with which they are supplied and any accessible pollen were removed, and the colonies were assigned and transferred to different sites (in urban and rural locations) in the East Sussex landscape (Table 1). The bumblebee nests assigned to urban sites were placed in gardens within sizeable urban/suburban areas (the smallest of which was Henfield, population ~5,000). Rural nests were placed on farmland, with less than 5% of the surrounding circle of 1 km radius comprised of gardens. No specific permission was required to carry out these studies and the bumblebee is not an endangered or protected species in the UK. The colonies were then left to develop for 10 weeks in the field, during which they were monitored and weighed every 2 weeks. At the end of the 10-week period, all surviving colonies were collected and immediately frozen at -80°C until dead. All queens, workers, males, and pupae were removed, counted and weighed, adult thorax width was measured (between the widest points at the base of the wing), and all individuals were stored at -20°C.

**Table 1:** The aluminium content of each pupa from each colony.

Colony ID	Dry Wt. g	[Al] μg/g	Colony Mean (SD) μg/g
1a	0.212	193.4	
1b	0.195	104.0	
1c	0.124	150.9	149.4 (44.7)
2a	0.431	24.5	
2b	0.399	28.7	
2c	0.368	35.6	
2d	0.492	28.1	29.2 (4.6)
3a	0.357	18.2	
3b	0.428	13.4	
3c	0.380	17.4	16.3 (2.6)
4a	0.341	37.9	
4b	0.205	64.5	
4c	0.331	49.9	50.8 (13.3)
6a	0.356	28.6	
6b	0.259	48.3	
6c	0.338	38.9	38.6 (9.9)
7a	0.355	33.4	
7b	0.403	29.7	
7c	0.282	41.1	
7d	0.309	28.2	33.1 (5.8)
13a	0.180	58.2	
13b	0.222	64.4	
13c	0.158	54.3	
13d	0.117	65.5	
13e	0.140	63.8	61.2 (4.8)
14a	0.327	19.8	
14b	0.257	15.9	
14c	0.131	44.8	
14d	0.160	33.8	
14e	0.176	38.3	30.5 (12.3)
15a	0.220	24.9	
15b	0.209	30.4	
15c	0.235	33.7	29.7 (4.5)
16a	0.130	69.3	
16b	0.180	60.4	
16c	0.154	80.3	
16d	0.145	80.0	72.5 (9.6)
18a	0.381	37.9	
18b	0.452	35.8	
18c	0.333	55.1	
18d	0.255	47.3	44.0 (8.9)
20.1a	0.195	41.0	
20.1b	0.193	53.5	
20.1c	0.184	52.0	48.8 (6.8)
24a	0.419	20.1	
24b	0.424	16.3	18.2 (2.7)
35a	0.176	72.4	
35b	0.107	42.6	
35c	0.194	63.3	59.4 (15.3)
39a	0.141	77.5	
39b	0.280	48.4	
39c	0.220	42.2	
39d	0.148	80.1	
39e	0.072	52.0	60.0 (17.5)
42a	0.516	31.5	
42b	0.546	21.5	
42c	0.266	27.2	26.7 (5.0)
44a	0.111	52.4	
44b	0.095	73.4	
44c	0.122	50.4	
44d	0.091	71.2	61.9 (12.1)
45a	0.111	39.1	
45b	0.097	40.8	
45c	0.188	29.7	
45d	0.222	24.6	
45e	0.136	42.1	35.3 (7.7)
51a	0.121	150.0	
51b	0.230	67.0	
51c	0.133	149.6	122.2 (47.8)
Q1a	0.241	38.9	
Q1b	0.293	33.4	
Q1c	0.253	39.7	33.7 (9.8)

### Aluminium measurements

Frozen pupae were allowed to thaw naturally in the laboratory in plastic Petri dishes. Each pupa was then subjected to a cleaning process which involved its immersion in 1 mM citric acid solution for 60 seconds followed by a subsequent immersion in ultra-pure water (conductivity < 0.067 μS/cm) for a further 60 seconds. The pupa was then blotted dry using filter paper and incubated at 37°C until it achieved a constant dry weight, usually taking 48–72 hours. Immersion in citric acid solution was used to remove any surface-associated aluminium and this procedure followed by the wash with ultra-pure water ensured that only tissue aluminium was measured in all subsequent analyses. Weighed pupae were then digested in a 1:1 mixture of 15.8M HNO_3_ and 30% *w/v* H_2_O_2_ in a microwave oven using an established method [9]. The resulting digests were transparent and made up to 20% HNO_3_ using ultra-pure water. Total aluminium in each digest was measured by transversely heated graphite furnace atomic absorption spectrometry (TH GFAAS) using a method developed in CE’s laboratory. Quality assurance data including the use of method blanks to account for incidental contamination have been published previously [9].

### Statistical analysis

Of the 20 colonies, the queens died prematurely in four. We tested the effect of site (urban or rural) and latitude on aluminium content found in the 20 bumblebee colonies (aluminium extracted from 2 to 5 pupae per colony). We further tested the response of peak colony weight, average worker/male/pupae weight, adult thorax width, and number of queens produced, to mean aluminium content, using only the 16 colonies in which the foundress queens survived. Data were analysed in R using Generalized Linear Models with quasipoisson errors (to account for under-dispersion of data) with measures of colony performance (peak colony weight; mean male weight; mean pupal weight; thorax size of workers, males, or queens; number of queens produced per colony) with mean aluminium content per nest, and the initial weight of each nest as covariates. Bonferroni corrections for multiple comparisons were applied.

## Results

Data were obtained on 2–5 pupae from 20 different colonies (mean ± SD; 3.6 ± 0.9). The aluminium content of individual pupae ranged from 13.4 to 193.4 μg/g dry wt. (Table 1). The mean (SD) aluminium content of all 72 pupae was 51.0 (33.0) μg/g dry wt. The aluminium content of pupae varied significantly from colony to colony with the range of the colony means (SD) being from 16.3 (2.6) for Colony 3 to 149.4 (44.7) μg/g dry wt. for Colony 1 (ANOVA, F_19,52_ = 13.5, p<0.001).

A greater, although not significant, amount of aluminium was found in urban colonies (mean 49.7 ± 34.2 SD) compared with rural (41.9 ± 19.3 SD). However, this result was due to a single colony, Colony 1, which was located in an urban garden in Uckfield (Table 2) and had an unusually high detected-level of aluminium for this dataset (note that colony 51 also had unusually high levels of Al, but was excluded from analyses as the queen died prematurely). Once this colony was removed, aluminium content in urban (42.5 ± 19.5 SD) and rural sites were similar. Due to this outlier, further analyses excluded Colony 1.

**Table 2:** The site (urban/rural) and grid references of 20 bumblebee colonies included in the experiment; mean aluminium content ± standard deviation; and initial colony weight, peak colony weight, mean worker, male, and pupal weight, and number of queens produced, as a measure of colony success.

Colony ID	Site	Grid Reference	Mean Aluminium Content ± SD	Initial Colony Weight	Peak Colony Weight	Mean Worker Weight	Mean Male Weight	Mean Pupal Weight	No. Pupae	No. Queens
			μg/g	g	g	g	g	g		Produced
24	Rural	50°56' N, 0°02' W	18.2 ± 2.7	486	641	0.197	0.167	0.538	28	7
42	Rural	50°52' N, 0°01' W	26.7 ± 5.0	471	723	0.142	0.308	0.504	28	3
15	Rural	50°54' N, 0°03' W	29.7 ± 4.5	480	728	0.058	0.121	0.398	115	0
35	Rural	50°57' N, 0°14' E	59.4 ± 15.3	489	576	0.15	0.146	0.253	18	5
39	Rural	50°56' N, 0°20' W	60.0 ± 17.5	492	488	0.162	-	0.342	9	3
Q1	Rural	50°54' N, 0°044' W	33.7 ± 9.8	467	-	-	-	-	-	-
45	Urban	50°52' N, 0°05' W	35.3 ± 7.7	479	-	-	-	-	-	-
20.1	Urban	50°52' N, 0°05' W	48.8 ± 6.8	488	-	-	-	-	-	-
51	Urban	50°52' N, 0°05' W	122.2 ± 47.8	492	-	-	-	-	-	-
3	Urban	50°56' N, 0°10' W	16.3 ± 2.6	469	703	0.191	0.169	0.604	54	1
2	Urban	50°56' N, 0°16' W	29.2 ± 4.6	511	603	0.197	0.294	0.672	53	4
14	Urban	50°46' N, 0°06' W	30.5 ± 12.3	496	731	0.094	0.196	0.282	31	0
7	Urban	50°57' N, 0°07' W	33.1 ± 5.8	481	1028	0.186	0.136	0.542	10	4
6	Urban	50°59' N, 0°05' W	38.6 ± 9.9	487	889	0.065	-	0.294	55	0
18	Urban	50°50' N, 0°09' W	44.0 ± 8.9	473	725	0.062	0.139	0.385	135	0
4	Urban	51°07' N, 0°029' W	50.8 ± 13.3	466	739	0.064	0.117	0.373	190	0
13	Urban	50°50' N, 0°13' W	61.2 ± 4.8	477	838	-	0.087	0.194	61	0
44	Urban	50°52' N, 0°00' W	61.9 ± 12.1	497	707	0.085	0.1	0.172	14	0
16	Urban	50°48' N, 0°20' W	72.5 ± 9.6	484	921	0.291	-	0.301	8	0
1	Urban	50°57' N, 0°06' E	149.4 ± 44.7	494	960	0.166	0.166	0.295	29	5

- data unavailable or not collected due to death of bumblebee queen early in experiment

**Fig 1:**
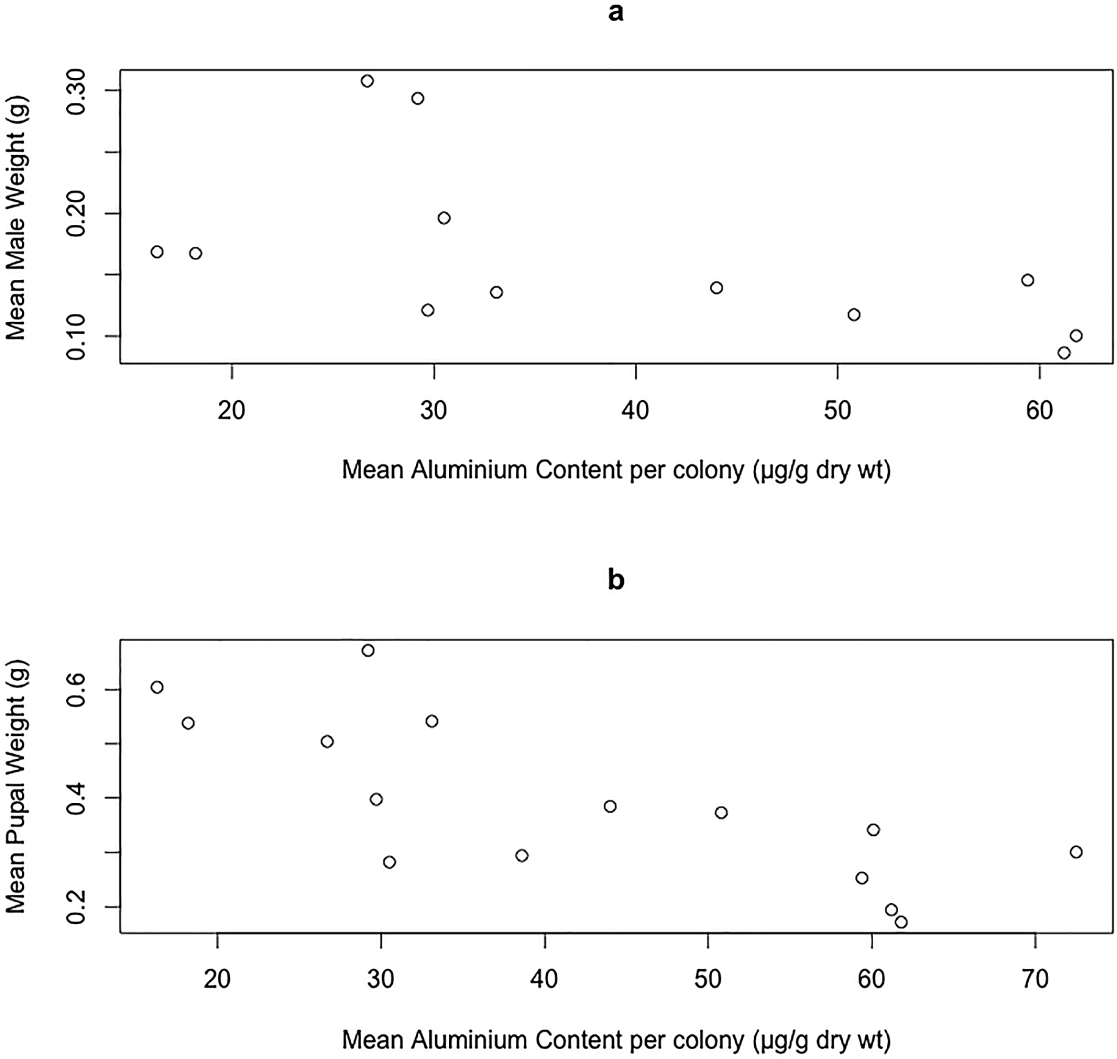
Scatterplot illustrating total aluminium content (μg/g dry wt.) found per pupae and average male weight (g) (a) and average pupal weight (g) (b) per colony.

Mean aluminium content per colony was a significant negative predictor of average male weight (P = 0.047 t = 2.307) and average pupal weight (P = 0.001, t = -4.128) (Fig 1, Table 3). If adjustment is made for multiple testing (seven response variables were explored) then the effect on male weight is no longer significant, but that of average pupal weight remains significant. No relationship was found between the level of aluminium found in pupae and peak colony weight, thorax size of workers, males, or queens, or the number of pupae or queens produced per colony. The initial weight of colonies was not a significant predictor of any of the response variables.

**Table 3:** Parameter estimates for Generalized Linear Models pupal and male weight responses to mean aluminium content.

	Parameter estimate ± SE	t-value	P-value
Mean Pupal Weight	-0.017 ± 0.004	-4.128	0.001
Mean Male Weight	-0.016 ± 0.007	-2.307	0.047

## Discussion

Bumblebee pupae were found to be heavily contaminated with aluminium. Judicious washing of pupae with citric acid solution and subsequent rinsing with ultra-pure water ensured the removal of any loosely associated aluminium from the surface of pupae and suggested that aluminium was either systemic or tightly associated with the surface of pupae.

The origin of the aluminium is likely to be pollen [7] and possibly nectar [8]. There are no other comparable data for aluminium content of bumblebees, pupae or adults, or indeed any other pupae of terrestrial insects. There are data for adult honeybees (*Apis mellifera*) and these indicate aluminium content between 4.6 and 15.5 μg/g dry wt. [10]. The range for the adult worker honeybees is considerably lower than for bumblebee pupae studied here and might reflect a difference between species, lifecycle stages or even methods for the measurement of aluminium in living tissues. Our data provide preliminary evidence that exposure to aluminium may be having an adverse effect on bumblebees, for colonies with high concentrations in the pupae tended to have smaller pupae. However, no other strong effects were observed, and our data set is small. We suggest that further investigation is needed, both to find out the generality and extent of exposure of pollinators to aluminium, and to determine the consequences.

Previously we have shown that sand flies fed aluminium-supplemented sucrose accumulated aluminium, specifically in their salivary glands [11]. We speculated that this aluminium might act as an inherent adjuvant in some forms of leishmaniasis and one might speculate further that aluminium in bees might be an unforeseen adjuvant in sensitisation to bee venom [12]. Aluminium has been measured in the tissues of *Drosophila melanogaster* fed aluminium in their diets though the values obtained are so low (0.015 to 0.047 μg/g dry wt.) that they are likely to be in error [13]. However, fruit flies fed aluminium in their diets exhibited acute toxicity as well as behavioural effects associated with locomotor activities and daily circadian rhythms suggesting possible neurotoxicity. Bees rely heavily on cognitive performance to navigate in their environment [14–16] select the most rewarding flowers [17] and avoid predators [18–19] It is conceivable that the high content of aluminium in bumblebee pupae measured here could interfere with the development or functioning of cognitive performance in adult bees. Aluminium is a known neurotoxin in humans [20] and brain aluminium content in excess of 3 μg/g dry wt. might be considered as pathological with possible contributions towards neurodegenerative disease including Alzheimer’s disease [21]. The observation here that the aluminium content of bumblebee pupae is an order of magnitude (or more) higher than levels harmful to humans gives cause for concern. Bee colonies are highly dependent on the ability of colony members to learn, navigate, and fly long distances during foraging, and so we might expect them to be particularly sensitive to neurotoxic effects. The neurotoxicity of metals in bees is largely unknown though a recent laboratory study has demonstrated the possible neurotoxicity of Zn(II) [22].
